# Improved Metabolite Prediction Using Microbiome Data-Based Elastic Net Models

**DOI:** 10.3389/fcimb.2021.734416

**Published:** 2021-10-25

**Authors:** Jialiu Xie, Hunyong Cho, Bridget M. Lin, Malvika Pillai, Lara H. Heimisdottir, Dipankar Bandyopadhyay, Fei Zou, Jeffrey Roach, Kimon Divaris, Di Wu

**Affiliations:** ^1^ Department of Biostatistics, Gillings School of Global Public Health, University of North Carolina at Chapel Hill, Chapel Hill, NC, United States; ^2^ Carolina Health Informatics Program, University of North Carolina at Chapel Hill, Chapel Hill, NC, United States; ^3^ Division of Pediatric and Public Health, Adams School of Dentistry, University of North Carolina at Chapel Hill, Chapel Hill, NC, United States; ^4^ Department of Biostatistics, School of Medicine, Virginia Commonwealth University, Richmond, VA, United States; ^5^ Department of Genetics, University of North Carolina at Chapel Hill, Chapel Hill, NC, United States; ^6^ Research Computing, University of North Carolina, Chapel Hill, NC, United States; ^7^ Department of Epidemiology, Gillings School of Global Public Health, University of North Carolina at Chapel Hill, Chapel Hill, NC, United States; ^8^ Division of Oral and Craniofacial Health Research, Adams School of Dentistry, University of North Carolina at Chapel Hill, Chapel Hill, NC, United States

**Keywords:** microbiome, metatranscriptome, metabolome, prediction, elastic net, random forest, metagenomics

## Abstract

Microbiome data are becoming increasingly available in large health cohorts, yet metabolomics data are still scant. While many studies generate microbiome data, they lack matched metabolomics data or have considerable missing proportions of metabolites. Since metabolomics is key to understanding microbial and general biological activities, the possibility of imputing individual metabolites or inferring metabolomics pathways from microbial taxonomy or metagenomics is intriguing. Importantly, current metabolomics profiling methods such as the HMP Unified Metabolic Analysis Network (HUMAnN) have unknown accuracy and are limited in their ability to predict individual metabolites. To address this gap, we developed a novel metabolite prediction method, and we present its application and evaluation in an oral microbiome study. The new method for predicting metabolites using microbiome data (ENVIM) is based on the elastic net model (ENM). ENVIM introduces an extra step to ENM to consider variable importance (VI) scores, and thus, achieves better prediction power. We investigate the metabolite prediction performance of ENVIM using metagenomic and metatranscriptomic data in a supragingival biofilm multi-omics dataset of 289 children ages 3–5 who were participants of a community-based study of early childhood oral health (ZOE 2.0) in North Carolina, United States. We further validate ENVIM in two additional publicly available multi-omics datasets generated from studies of gut health. We select gene family sets based on variable importance scores and modify the existing ENM strategy used in the MelonnPan prediction software to accommodate the unique features of microbiome and metabolome data. We evaluate metagenomic and metatranscriptomic predictors and compare the prediction performance of ENVIM to the standard ENM employed in MelonnPan. The newly developed ENVIM method showed superior metabolite predictive accuracy than MelonnPan when trained with metatranscriptomics data only, metagenomics data only, or both. Better metabolite prediction is achieved in the gut microbiome compared with the oral microbiome setting. We report the best-predictable compounds in all these three datasets from two different body sites. For example, the metabolites trehalose, maltose, stachyose, and ribose are all well predicted by the supragingival microbiome.

## Introduction

The importance of the human microbiome in health and disease is undeniable; site-specific microbial communities interact both with the environment and the host and influence numerous biological processes (Wilkinson et al., 2021). Aside from the logical interest in understanding the composition of the microbiome (Tsilimigras and Fodor, 2016) (i.e., relative abundance of identified taxa), measuring and understanding its associated metabolic activities are arguably of utmost biological relevance. Recent studies have linked the metabolome with several important health conditions including inflammatory bowel disease (IBD) (Lloyd-Price et al., 2019), obesity and type II diabetes (Canfora et al., 2019), cholesterol levels (Kenny et al., 2020), and early childhood dental caries (ECC) (Heimisdottir et al., 2021). Despite the rapidly increasing availability of microbiome data in large health cohorts, metabolomics data are still scant. This is an important limitation because the lack of, or considerable missingness of, metabolite information in microbiome studies can diminish their potential in inferring functions and important biological targets.

It follows that methods that help fill in the functional information gaps in microbiome studies are valuable and necessary. Because “matched” microbiome and metabolome datasets are extremely scant, most current methods rely on metabolic pathway inferences from taxonomic and metagenomic data, such as in the HMP Unified Metabolic Analysis Network (HUMAnN) (Franzosa et al., 2018). While the value of this approach is well-documented for the analysis of some microbial consortia (e.g., the human gut) (Lloyd-Price et al., 2019; Thomas et al., 2019), HUMAnN cannot make predictions for individual metabolites. Moreover, its accuracy has not been benchmarked and its performance in other microbial communities with distinct ecology and function (e.g., the oral cavity) remains unknown. This is important because metabolomes measured at different body sites may include, besides the products of microbial metabolism, biochemical contributions from the host and the environment [e.g., dietary sugars in the study of dental biofilm (Heimisdottir et al., 2021)]. Although an accurate determination of metabolite sources may not always be possible, predictions of these biofilm metabolites using microbiome information are highly desirable.

Along these lines, in 2016, Noecker and colleagues (Noecker et al., 2016) added to the available analytical toolbox by leveraging 16S rRNA data. Their method enabled model-based integration of metabolite observations and species abundances using taxonomy and paired metabolomics data from ~70 vaginal samples. More recently, MelonnPan (Mallick et al., 2019) was developed to obtain metabolomic profiling of microbial communities using amplicon or metagenomic sequences. This new method was motivated by and applied in the context of paired microbiome and metabolome data in the context of an IBD cohort. The motivation for the present new method development is to improve existing analytical approaches available for metabolite prediction and functions using microbiome data (Sanna et al., 2019). To this end, we leverage existing microbiome and metabolome data from a study of early childhood oral health (ECC study) and two IBD studies of the human gut microbiome. The elastic net model (ENM, used in MelonnPan), compared to LASSO or ridge regression, benefits from keeping both the singularities at the vertices, which is necessary to accommodate data sparsity, and the strict convex edges for grouping among correlated variables.

Inspired by MelonnPan and MIMOSA, we propose an improved prediction method for individual metabolites using microbiome information in the same (i.e., matched or paired) biological samples, called “elastic net variable importance model (ENVIM)”. ENVIM improves upon ENM algorithms by weighting microbial gene family features using random forest variable importance (VI) to enhance the contribution of most prediction-informative genes. ENVIM outputs predicted metabolites from matched microbiome samples, as well as gene families and their weights informing metabolite prediction.

In this paper, we present the development, application, and evaluation of ENVIM. We compare it against MelonnPan in three datasets generated from oral and gut samples, so that we can also compare metabolite predictive performance between different body sites. The predictors can be three different gene family data types: metagenome only, metatranscriptome only, and the combination of both metagenome and metatranscriptome data. The top predictable compounds have been reported in these three datasets from two different body sites. To quantify the taxonomic and functional relationship of the most prediction-contributing microbial gene families in ENVIM, an enrichment analysis is performed and several predictive gene families are detected in species of the oral biofilm.

## Material and Methods

### Cohorts and Data Description

In the following section, we describe the microbiome and metabolome data used for the new method development and application, alongside the three contributing studies.

#### ZOE 2.0 Study Data

ZOE 2.0 is a community-based molecular epidemiologic study of early childhood oral health in North Carolina (Divaris et al., 2020; Divaris and Joshi, 2020). The study collected clinical information on preschool-age children’s (ages 3–5) dental cavities (referred to as early childhood caries or ECC) (Ginnis et al., 2019) and supragingival biofilm samples from a sample of over 6,000 children (Divaris et al., 2019). A subset of participants’ biofilm samples underwent metagenomics, metatranscriptomics, and metabolomics analyses, under the umbrella Trans-Omics for Precision Dentistry and Early Childhood Caries or TOPDECC (accession: phs002232.v1.p1) (Divaris et al., 2020). As such, metagenomics (i.e., shotgun whole-genome sequencing or WGS), metatranscriptomics (i.e., RNA-seq), and global metabolomics data (i.e., ultra-performance liquid chromatography-tandem mass spectrometry) (Evans et al., 2009; Evans et al., 2014; Heimisdottir et al., 2021) from supragingival biofilm samples of ~300 children, paired with clinical information on ECC, are available. After exclusions due to phenotype and metabolite missingness described in a previous publication (Heimisdottir et al., 2021), the joint microbiome–metabolome data include 289 participants. There are 503 known metabolites included in the ZOE 2.0 dataset. Metagenomics and metatranscriptomics data in reads per kilobase (RPK) were generated using HUMAnN 2.0. Here, we use species-level (205 species), gene family (403k gene families), pathway (397 pathways), and metabolome (503 metabolites) data.

#### Lloyd-Price Study Data

The Lloyd-Price dataset (Lloyd-Price et al., 2019) was obtained from the IBD multi-omics database (https://ibdmdb.org). It is derived from a longitudinal study that sought to generate profiles of multiple types of omics data among 132 participants for 1 year and up to 24 time points. Several different types of omics data of the study include WGS shotgun metagenomics, RNA-seq metatranscriptomics, and metabolomics. The corresponding metadata include demographic information such as occupation, education level, and age. These gut microbiome data are in counts per million (CPM) and were derived using functional profiles 3.0 in HUMAnN 3.0. For this study, we merged data of individual gene families for 1,638 samples for 130 subjects and individual metatranscriptomics gene families for 817 samples for 109 subjects, respectively. The merged metagenomics gene family data include about 2,741k gene families and 1,580 samples. Merged metatranscriptomics gene family data include about 1,079k gene families and 795 samples. The metabolomics data were generated using four liquid chromatography tandem mass spectrometry (LC-MS) methods including polar compounds in the positive and negative ion modes, lipids, and free fatty acids and bile acids and include 81,867 metabolites in 546 samples for 106 subjects. Most metabolites have not been annotated into known biochemicals and, thus, were excluded from prediction. After limiting the dataset to known metabolites and removing “redundant ions” in “HMDB” ID, there remained 526 metabolites to be predicted.

#### Mallick Study Data

The Mallick data comprised the main real-life dataset used in the development of the MelonnPan method (Mallick et al., 2019). These gut microbial data (WGS shotgun sequencing and metabolomics) were collected from two cross-sectional IBD cohort studies, namely, the Prospective Registry cohort for IBD Studies at the Massachusetts General Hospital (PRISM, with 155 subjects) and the Netherlands IBD cohort (NLIBD, with 65 subjects). Therefore, they comprise two independent cohorts of subjects. The raw data were obtained through a combination of shotgun metagenomic sequencing and the same four LC-MS methods (Franzosa et al., 2019) as in the Lloyd-Price study. Gene family data in RPK units were derived using HUMAnN 2.0 and normalized to reads per kilobase per million sample reads (RPKM). The raw metagenomics gene family dataset includes one million gene families. The investigators (Mallick et al., 2019) filtered out genes with low abundance and prevalence resulting in a processed dataset of 811 gene families available in the R package *MelonnPan* (melonnpan.training.data and melonnpan.test.data) for 222 total subjects. The microbiome data have been preprocessed and normalized into relative abundance. The metabolite abundance data (8,848 metabolites and 220 subjects) have been made available by Franzosa et al. (2019). Those authors used 466 metabolites for analyses, a subset that was confirmed experimentally against laboratory standards prior to application in *MelonnPan*. In the present study, we use information from these 466 metabolites to compare the power of the new ENVIM method against *MelonnPan*. To accomplish this, we normalized the metabolite abundance data for all 8,848 metabolites into relative abundance (compositional format, obtained *via* dividing the normalized abundance by the sample-level total normalized abundance). Among them, we used the same 466 metabolites with laboratory standards as selected in the paper of *MelonnPan* (Mallick et al., 2019). Data missingness is not an issue in the Mallick metabolome data.

### Metabolomics Data Preprocessing and Normalization

**Figure 1:**
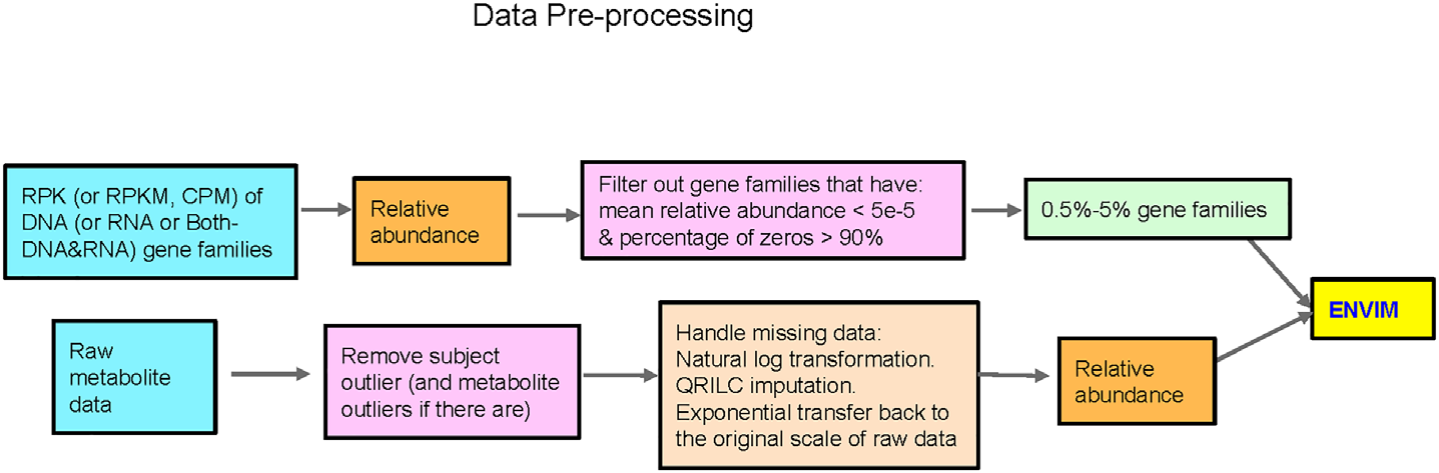
Flowchart of data preprocessing in microbiome and metabolome. QRILC was not used for the Mallick data, but was used for the ZOE 2.0 and Llloyd-Price data. Metabolites that have percentage of NA > 90% will also be removed before handling missing data.

An overview of the approach for metabolome data is presented in **Figure 1** and elaborated in detail below.

#### Metabolomics Missing Data Imputation: ZOE 2.0 and Lloyd-Price Studies

In ZOE 2.0, 87% of metabolites have some missing data, whereas 58% have missing values in Lloyd-Price. To address missingness in these two cohorts, we applied a rigorous feature-wise quantile regression imputation of left-censored data (QRILC) (Wei et al., 2018) to impute missing metabolite values and avoid underestimated metabolite-level variance, as in a previous publication (Heimisdottir et al., 2021). Each of the 289 included participants has <90% missing data across the 503 metabolites in ZOE 2.0. We applied a similar preprocessing filter for the Lloyd-Price data (i.e., removing outlier subjects, **Supplemental Figure 1**), resulting in the exclusion of 15 outlier subjects with the largest numbers of missing metabolite values, as well as outlier metabolites with >90% missing values. Consequently, we proceeded to analyze 522 metabolites in 531 samples from the Lloyd-Price data.

The QRILC imputation method was applied after a natural log data transformation, and the imputed data were exponentiated to back transform the data to RPK (in ZOE 2.0) or CPM (in Lloyd-Price) scales. Because MelonnPan requires metabolite data to be inputted as compositional, we converted RPK and CPM imputed data to a compositional format before predictive modeling.

#### Metabolites Filtered by Metabolic Pathways (ZOE 2.0, Lloyd-Price, and Mallick)

We used the MetaCyc database to retain only “reactive” metabolites (Caspi et al., 2014). To achieve this, we considered the membership of the metabolites in any MetaCyc metabolic pathway, reflecting reactions between bacteria and metabolites, and carried out the following steps:

1. In the MetaCyc database, we identify metabolites in each of the pathways predicted by both metagenomics and metatranscriptomics data in Functional Profile 2.0 generated by HUMAnN 2.0 (ZOE 2.0) and Functional Profile 3.0 generated by HUMAnN 3.0 (Lloyd-Price data). Of note, no pathway information exists in the available Mallick metagenomics and metatranscriptomics data.
2. We used metabolite labels (KEGG ID, HMDB, PubChem, and metabolite name, provided in Metabolome data annotation, provided by the manufacturer) in each of the three datasets, as the mapping IDs for each metabolite.
3. In MetaCyc, regardless of the metabolite label, only one unique MetaCyc “weblink” or universal mapping id is returned if the metabolite is in the database. This way, reactive metabolites identified in step 1 can be matched with metabolites identified in step 2. 3) Therefore, we identify metabolites that are in the observed pathways. Finally, we filter out metabolites with low abundance (metabolites with mean relative abundance <10^−4^) and low prevalence (metabolites with percentage of zeros in >90% of the samples). Consequently, there were 149 metabolites in pathways in ZOE 2.0, 125 in Lloyd-Price, and 251 in the Mallick data. Metabolites in Mallick data only have been filtered by the abundance, without being filtered by metabolic pathways. To compare the prediction of metabolites in pathways with the prediction of all metabolites, we considered both sets of metabolites in our analyses.

### Microbiome Data Preprocessing and Normalization

An overview of the approach for microbiome data is presented in **Figure 1** and elaborated in detail below. First, we matched gene family-level microbiome data with metabolome data by participant or sample unique identifier. Then, the scaled (RPK, RPKM, or CPM) gene family abundances were converted to compositional data, relative to the total scaled gene family abundances within a sample. Then, we filtered out gene family features with low relative abundance (mean relative abundance <5 × 10^−5^) and low prevalence (percentage of zeros in >90% of the samples) and thus kept 0.5%–5% of gene family features. The same procedures were performed for both metatranscriptomics (briefly referred to as “RNA” thereafter) and metagenomics data (briefly referred to as “DNA” hereafter). When both DNA and RNA data (briefly as “Both” hereafter) are considered predictors, a gene name may correspond to two “gene features,” one for each data type. The same data preprocessing and normalization procedures were followed for the three cohorts, with sample sizes and feature numbers presented in **Table 1**. To prevent overfitting when evaluating ENM and ENVIM, we divided samples into training (75% of subjects) and testing datasets (25% of subjects).

**Table 1:** Sample size and number of selected gene family features.

		Training genes	Testing genes	Genes in both	Subjects	Metabolites	Metabolites (in pathways)
ZOE 2.0	DNA (total 403k genes)	1,355	1,276	1,214	289	503	149
	RNA (total 403k genes)	1,805	1,826	1,667	287	503	149
	Both (total 806k genes)	3,158	3,183	2,948	287	503	149
Lloyd-Price	DNA (total 2,741k genes)	726	712	633	359	522	125
	RNA (total 1,079k genes)	726	704	600	282	522	125
	Both (total 3,820k genes)	1,424	1,508	1,211	269	522	125
Mallick	DNA (total 1,000k genes)	811	811	811	220	466	251 (filter only)

Testing genes: genes that can be used in the testing set. Training genes: genes that can be used in the training set. Genes in both: genes that are in both training and testing sets.

### The Existing ENM Method for Microbiome Data-Based Metabolite Prediction

As mentioned previously, the existing method available for predicting metabolite abundance using metagenomics data is MelonnPan (Mallick et al., 2019) (Model-based Genomically Informed High-dimensional Predictor of Microbial Community Metabolic Profiles). In this study, in MelonnPan, we used all filtered metagenomic gene family features in the 10-fold cross-validated elastic net model (ENM) (Zou and Hastie, 2005) to predict metabolite abundance (**Equation 1**).

However, using all filtered metagenomic gene family features in the model may dilute the effect of some important gene family features contributing to the prediction of metabolite abundance. This limitation can be improved upon, and therefore, in this paper, we set out to improve the ENM and develop a new algorithm.

The MelonnPan software was downloaded from GitHub (https://github.com/biobakery/melonnpan) or in *MelonnPan* package in R, and the CSV output files “Predicted_Metabolites.txt” (Train) and “MelonnPan_Predicted_Metabolites.txt” (Test) are used as the prediction results of MelonnPan.

The ENM assumes the model,


$$y_{i}=x_{i}^{'}\;\beta +\varepsilon_{i},$$

where *β* = (*β*_0_ *, β*_1_ *, … , β_p_*)*'* and $\hat{\beta }$ , the ENM estimator of *β*, is found by minimizing the objective function of ENM,


Equation 1 $$L_{ENM}=\frac{1}{2N}\sum_{i=1}^{N}{\left(y_{i}-x_{i}^{'}\beta \right)}^{2}+\lambda \sum_{j=1}^{p}\left\{\frac{1-\alpha }{2}\beta_{j}^{2}+\alpha \left|\beta_{j}\right|\right\}.$$

### Evaluation Methods

Following Cohen’s criterion (Cohen, 1988), by which a correlation coefficient of 0.3 is considered to be the median size, we define well-predicted (WP) metabolites as those with Spearman correlation ≥0.3, and those with correlation <0.3 as poorly predicted. This criterion has also been used in the development of MelonnPan (Noecker et al., 2016). We evaluated the predictive performance of the new method ENVIM by comparing it against MelonnPan. Additionally, we compared Spearman correlations and mean square error (MSE) between the predicted and observed metabolites in both the training stage and the testing stage for all three datasets and both methods.

## Results

### The Improved ENM Based on Variable Importance Score (ENVIM)

#### Algorithm and Procedure in ENVIM

**Figure 2:**
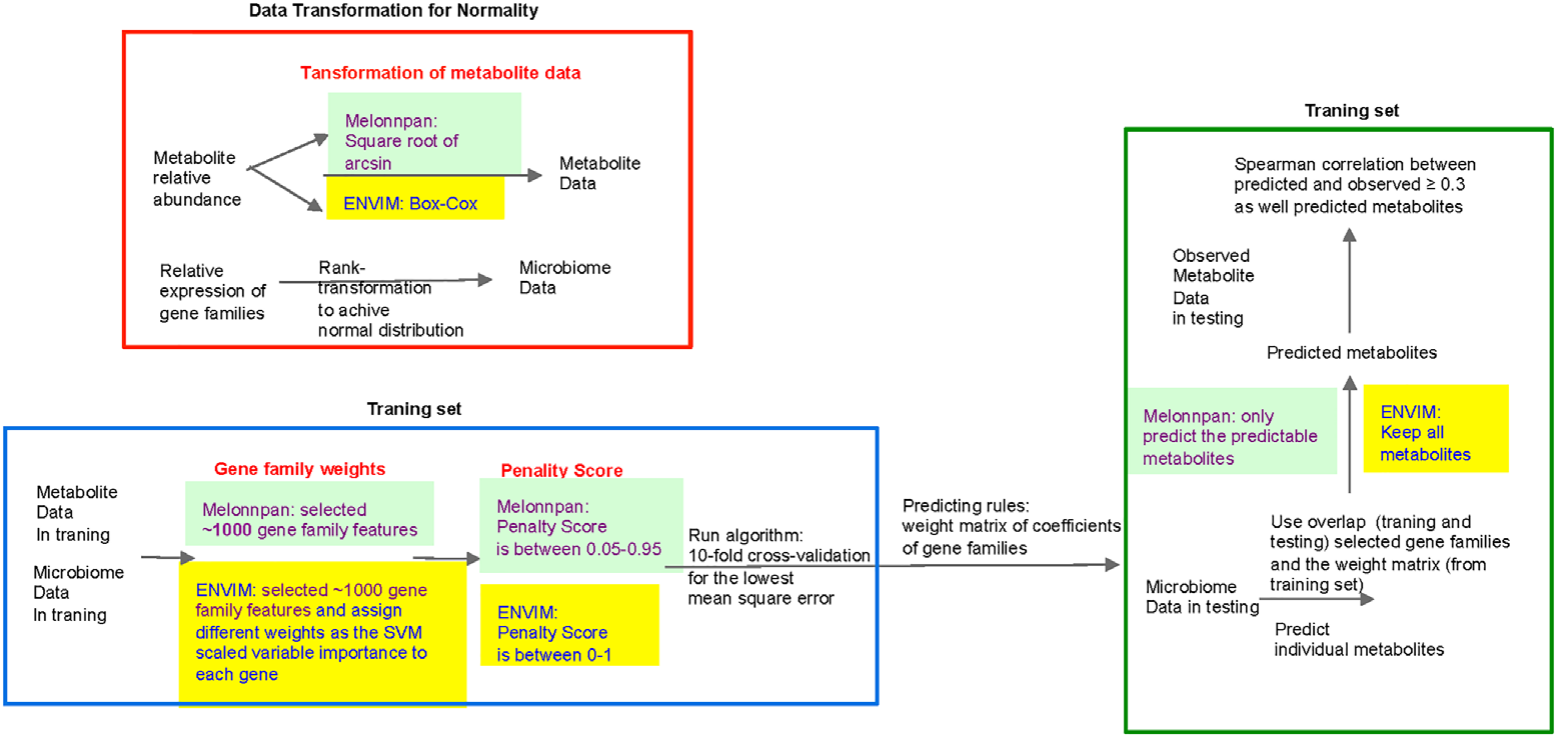
Flowchart of MelonnPan and the elastic net variable importance model (ENVIM). The three differences between them include (red text) 1) transformation of metabolite data, 2) gene family weights, and 3) penalty score. The predictable metabolites are defined as the metabolites that have a significant Spearman correlation with the adjusted *q*-value (testing whether the correlation is zero) below the default threshold in the training set.

The new algorithm ENVIM (**Equation 2**) was developed by extending the existing ENM with the random forest-derived variable importance to enhance the weights of important features in the prediction. ENM was previously used in the MelonnPan framework for microbiome-based metabolome prediction. The procedure in ENVIM and the comparison between ENM and ENVIM are shown in **Figure 2**. Because ENM assumes the normality of the error term, and there are typically excess zeros, skewness, and extreme values in metagenomics and metatranscriptomics data, we rank-transform gene family features in each sample to a normal distribution by using the *rntransform* (Aulchenko et al., 2007) function in the R package *GENABEL* for training data and testing data separately. The training metabolite abundance data are transformed to a normal distribution using a Box–Cox transformation. After fitting the model in the training data, predicted metabolite abundances are transformed back to relative abundances with γ determined by the training metabolite abundance data.

Including all gene families into the model could make the cross-validated MSE larger, whereas including only a small part could make the error larger. Therefore, to identify a model with minimum cross-validated error, one needs to iterate different numbers of gene families. Because we prioritize gene families with high importance relative to metabolites, we use a non-linear regression model to determine the importance of gene families for each metabolite. We train a cross-validated random forest model (Breiman, 2001) by using the training data and use the *varImp* function in the *caret* package (Kuhn, 2008) in R to find the scaled importance score (0–100) between each independent feature and the metabolite abundance. We introduce a unique step that uses the scaled variable importance scores to define sets of the top gene families according to a predefined set of thresholds, for example, 90, 80, 70, etc. We use the *glmnet* (Friedman et al., 2010) package in R to run cross-validated ENM and choose penalty parameters for each model.

In the *training stage*, we assign the importance score from 0 to 100 in 10 cumulative intervals (90–100, 80–100,…, 10–100, 0–100) and remove the intervals without gene families. In the ENM, we consider gene families as the independent variables and metabolite abundances as the dependent variables. We consider different sets of gene families with different importance scores. For each set of gene families, we conduct a 10-fold cross-validated ENM and build 10 models with different values of the tuning parameter γ, ranging from 0 to 1. For each model, we measure the MSE between the measured metabolite abundance and the predicted values to determine the best model (i.e., the model with the lowest MSE). To maintain reproducibility, we maintain the same random seed and permute the same fold index number in the ENM. The matrix of regression coefficients of gene families from the best model identified in the training set will be output as a weight matrix.

In the *testing stage*, for the prediction of each metabolite, we use the weight matrix output from the training stage for prediction, if the gene families are also detected in the testing set. Because we have transformed the compositional metabolite abundance to a normal distribution using a Box–Cox transformation in the training stage, we transform the predicted metabolite abundance data back to the original compositional scale based on γ calculated in the training step.

ENVIM assumes the following model:


$$y_{i}=x_{i}^{'}\;\beta +\varepsilon_{i},$$

where *β* = (*β*_0_ *, β*_1_ *, … , β_p_*)*'*, and ${\hat{\beta }}^{ENVIM}=\arg\min_{\beta }\underset{k\in K}{\min}L_{ENVIM}(k)$ , the ENVIM estimator of *β*, is found by minimizing over *k* and *β* the objective function,


Equation 2 $$L_{ENVIM}(k)=\frac{1}{2N}\sum_{i=1}^{N}{\left(y_{i}-x_{i}^{'}M_{k}\beta \right)}^{2}+\lambda \sum_{j=1}^{p}s_{k,j}\left\{\frac{1-\alpha }{2}\beta_{j}^{2}+\alpha \left|\beta_{j}\right|\right\}.$$

Here we define *VI_j_* as the variable importance score for the *j*th variable given by a random forest; $S_{k}={\left\{S_{k,j}\right\}}_{j}^{p}=I{\left\{VI_{j}\geq k\right\}}_{j=1}^{p}$ is the variable selection indicator vector, which is 1 if the importance score for the *j*th variable is larger than the threshold *k*; $M_{k}=diag\{(1,S_{k}^{'})\}$ is the corresponding diagonal variable selection matrix that includes the intercept term; and *K* is a set of the candidate *k* values. *K* is defined adaptively so that it covers the range of the variable importance scores reasonably. In our analysis, we set *K* = {0, 10, 20, …, 90}.

#### Three Key Differences Between MelonnPan and ENVIM for Predicting Individual Metabolites

1. Transformation of metabolite abundance data into a normal distribution

**Figure 3:**
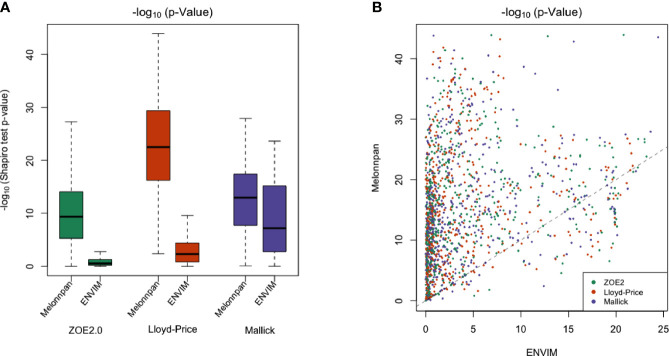
**(A)** Boxplot of −log_10_ of Shapiro–Wilk test *p*-values to test the normality of transformed relative metabolite abundances in all three data applied with Box–Cox transformation (ENVIM used) and arcsine square root transformation (MelonnPan used). **(B)** Scatter plot for comparing −log_10_ of *p*-values from the Shapiro–Wilk test (normality) between Box–Cox transformation (*x*-axis) and arcsine sqrt (*y*-axis) transformation. Almost all of the points are above the *y* = *x* line, which indicates that the −log_10_ of *p*-value after Box–Cox transformation is smaller than after arcsine sqrt transformation and normality after Box–Cox transformation is better. Each point is one metabolite.

MelonnPan transforms relative metabolite abundances with the arcsine square root operator, whereas we use a Box–Cox transformation in ENVIM. To test the normality of the transformed data, we compare the *p*-values of the Shapiro–Wilk test statistics for both the Box–Cox (**Equation 3**) and the arcsine square root transformations of metabolite relative abundances. The Shapiro–Wilk test is typically used for examining distribution normality for a continuous variable. The smaller the *p*-values, equivalently, the larger the −log_10_(*p*-values) are, the more evidence the data are not normally distributed. Overall, the −log_10_(*p*-values) from the Box–Cox transformation in ENVIM are smaller than those from the arcsine square root transformation (**Figure 3A**), which indicates that the Box–Cox-transformed data are more normally distributed. In addition, the Box–Cox transformation yields better normal approximation than the arcsine square root transformation for most of the metabolites (**Figure 3B**).

Box–Cox transformation


$$y^{\prime }=\{\begin{array}{l} \frac{y^{\omega }-1}{\omega },\omega \neq 0\\ \log(y),\omega =0 \end{array},$$

where *y* is the relative abundance, and *y*′ is the transformed abundance.

**2.** Different sets of gene families are carried forward to the prediction model

MelonnPan uses all gene families in the training data in the ENM and ultimately predicts metabolites in the testing stage using the same features. However, regressing against all gene families may dilute the effect of important gene families. Thus, unlike MelonnPan, we use a variable importance criterion to select different sets of gene families and include them in the prediction models.

**3.** The range of *α* values in ENM

Alpha (*α*) is the weight between the L_1_ and L_2_ penalty terms in the ENM, and in combination with γ values, the set of values that minimizes the 10-fold cross-validated MSE (**Equation 1**) is chosen. When *α* is 0, the model reduces to a Ridge regression model which has the advantage of dealing with highly correlated independent variables; when *α* is 1, the model becomes a Lasso regression model which has a variable selection capacity; when *α* is between 0 and 1, the model includes the advantages of both Ridge regression and Lasso regression. In MelonnPan, the range of *α* values does not include 0 and 1, which excludes either the Ridge or Lasso regression models, and it may not consider variables with high importance. The range of *α* in our ENVIM includes 0 and 1. By allowing a larger range of *α*, we can include the Ridge regression model as the potential final model, which does not unduly exclude variables with high importance.

The ENVIM software written in R statistical language is available in GitHub (https://github.com/jialiux22/ENVIM). The *ENVIM_predict* function is for metabolite prediction only, and the *ENVIM* function for both the metabolite prediction and the evaluation of the observed metabolomics dataset in the testing set is also available. Both R functions will output the weight matrix between gene families and metabolites. The weight matrix in testing has the same values as in training if they have the same number of gene families. Some contributing gene families in the weight matrix of the training set may not be measured in the testing set, so the weight matrix used by the testing set includes only the gene families that are shared by both the training and the testing sets.

### Method Comparison for Prediction of Individual Metabolites in the Three Datasets

#### Correlation-Based Method Comparison for All Metabolites

We used microbial gene family data to predict individual metabolites in the matched samples (that are from the same biological sample in that one proportion is for microbiome and the other is for metabolome). We compared the prediction results between ENVIM and MelonnPan, in terms of Spearman correlation and MSE between predicted and observed values of each of the metabolites, in three datasets (ZOE 2.0, Mallick data, and Lloyd-Price data) at each of the three data modalities of microbial gene families, i.e., DNA-seq, RNA-seq, and Both (RNA and DNA). The MSE in the testing set is used for comparison between the methods (**Supplemental Figure 2**).

**Figure 4:**
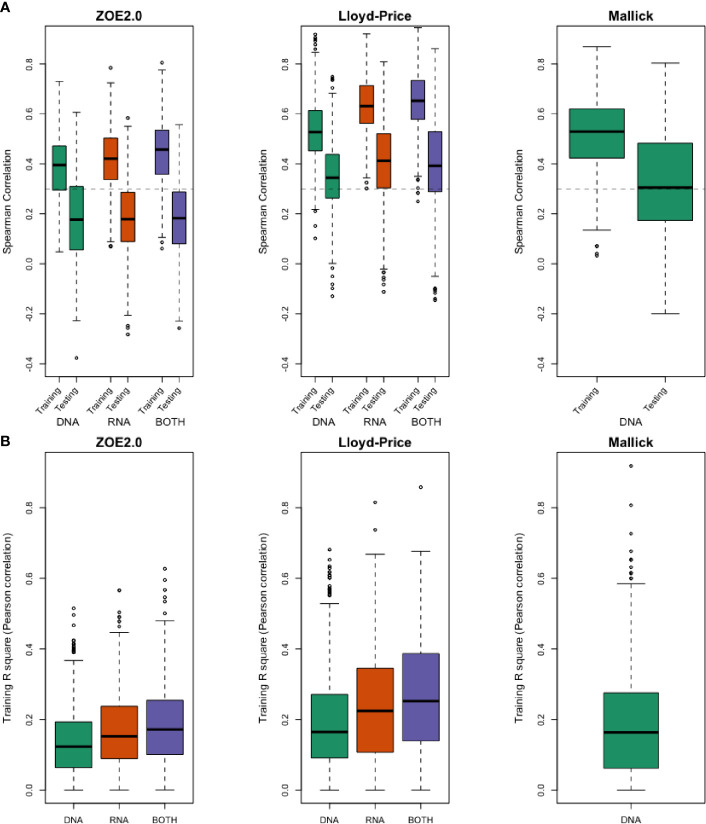
**(A)** Evaluation using Spearman correlation *r* in training stage and testing stage between predicted values and the observed values by using DNA-seq data only, RNA-seq data only, and both for ZOE 2.0 data, Lloyd-Price data, and Mallick data. **(B)** *R*-square in the training stage, as the percentage of variance explained by prediction models to demonstrate the lack of overfitting.

We have summarized the prediction results (**Table 2** and **Figure 4A**) for all metabolites in terms of Spearman correlation (*r* = 0.3) according to three aspects: method comparison, data modality comparison, and microbial community (i.e., body site) comparison. Overall, in method comparison, ENVIM produces higher percentages of well-predicted metabolites than MelonnPan in all three datasets, in both testing and training sets, and for DNA, RNA, and Both when available (**Table 2**).

**Table 2:** Prediction results (first four columns of numbers) in terms of Spearman correlation for all metabolites to be predicted.

	Training (ENVIM)	Training (MelonnPan)	Testing (ENVIM)	Testing (MelonnPan)	Predictable metabolites (defined by MelonnPan)
ZOE 2.0 (NM = 503)					
DNA only	356 (71%)	63 (13%)	**124 (25%)**	47 (9%)	70
RNA only	409 (81%)	157 (31%)	106 (21%)	68 (14%)	163
Both DNA and RNA	423 (84%)	146 (29%)	110 (22%)	73 (15%)	154
Mallick cohort (NM = 466)					
DNA only	408 (88%)	239 (51%)	225 (48%)	178 (38%)	249
Lloyd-Price cohort (NM = 522)					
DNA only	501 (96%)	271 (52%)	322 (62%)	193 (37%)	305
RNA only	521 (100%)	298 (57%)	**393 (75%)**	236 (45%)	318
Both DNA and RNA	518 (99%)	306 (59%)	381 (73%)	232 (44%)	323

Based on the “well-prediction” criterion, defined as Spearman correlation ≥0.3 between the observed and the predicted metabolites, the numbers of well-predicted metabolites with different prediction methods, datasets, and modality levels (DNA, RNA, and Both) are presented for comparing MelonnPan and ENVIM. NM is the number of metabolites to be predicted. Percentages in parentheses (%) represent the number of well-predicted metabolites divided by the total number of metabolites (NM) to be predicted in each study. The Mallick cohort has only metagenomics data available. The last column presents numbers of “predictable metabolites,” defined by MelonnPan, also seen in the **Figure 2** legend. Bold in the column of in testing results represents the highest number of well-predicted metabolites among the three modalities (DNA, RNA, both DNA and RNA) in the ZOE2.0 cohort and the Lloyd-Price cohort.

Generally, in data modality comparison, RNA gene family data produce higher percentages of well-predicted metabolites than DNA data. In the Lloyd-Price study, RNA-only data typically give higher percentages of well-predicted metabolites. In the ZOE 2.0 and Lloyd-Price data, both DNA and RNA predictors produce similar percentages but are not always superior to the DNA-only or RNA-only data-based predictors. However, results from both DNA and RNA predictors are never the worst. Unsurprisingly, the well-predicted percentage of metabolites in testing sets is lower than in the training set (**Table 2**). The boxplots of Spearman correlations between the predicted and observed metabolites for all metabolites (**Figure 4A**) suggest that the correlations between the ENVIM-predicted and the observed metabolites are higher in RNA than in DNA, but are comparable to correlations in both DNA and RNA. We are aware that in the testing sets, MelonnPan only outputs the predictable metabolites (defined as well-predicted metabolites in the training set, the last columns in **Table 2**), so it is not as appropriate for MelonnPan, as compared with ENVIM, to calculate the Spearman correlation distribution for all metabolites in **Figure 4A**. It must also be noted that the highest proportion of well-predicted metabolites is found in the two gut microbiome studies (Lloyd-Price study and Mallick study), and the lowest is in the supragingival dental biofilm (ZOE 2.0 study) (**Table 2**). Since Spearman correlation in both the Lloyd-Price and Mallick datasets is higher than that in ZOE 2.0 (**Figure 4A**), it is reasonable to suggest that metabolite prediction is better in gut microbial communities than in the oral microbial communities.

**Figure 5:**
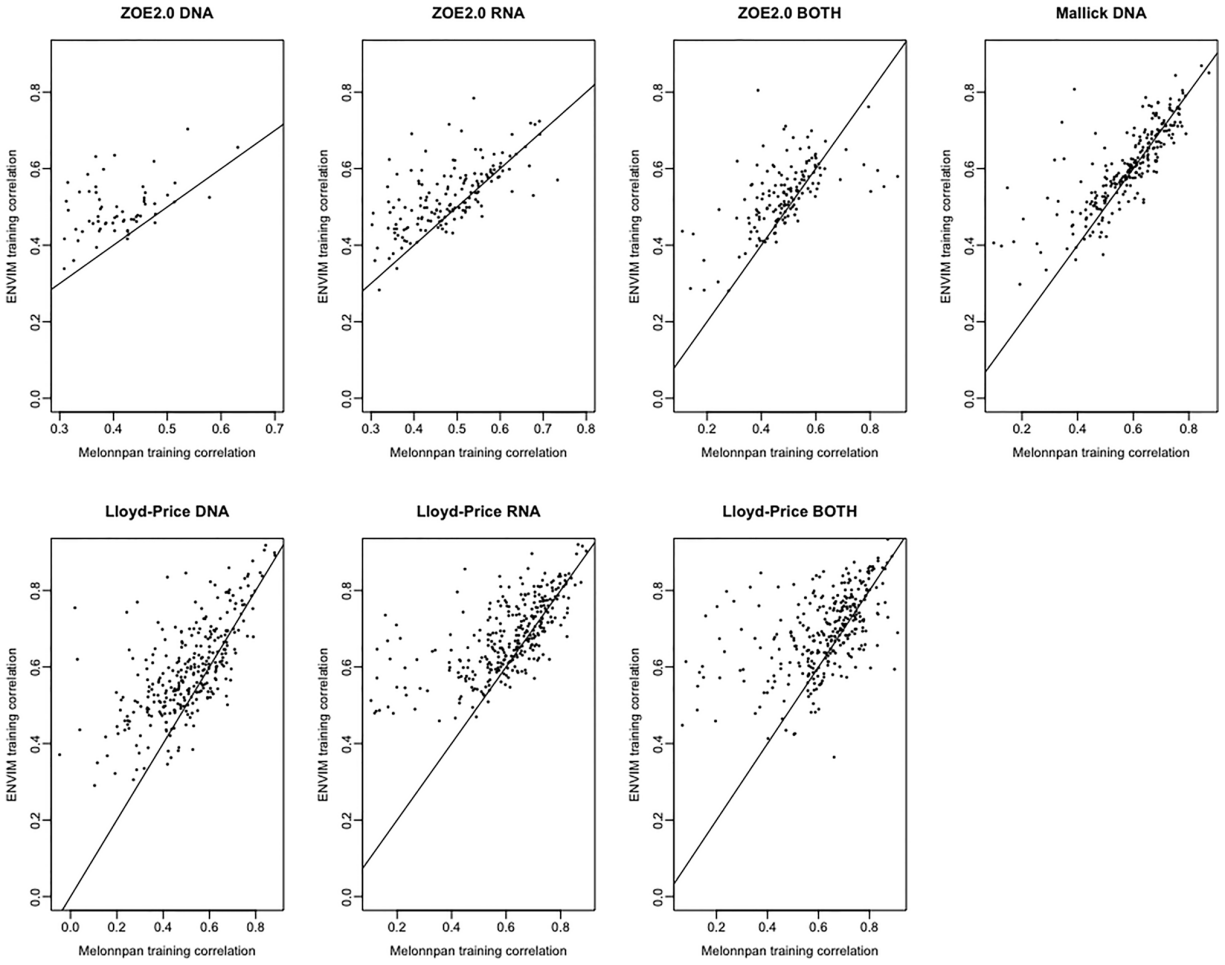
For DNA, RNA, and both in each study and the training set, this shows the scatter plot of Spearman correlation in ENVIM (*y*-axis) and MelonnPan (*x*-axis). Spearman correlation is based on observed metabolite abundance and predicted values. If our calculated correlation is NA, the metabolites will be not included in this figure.

**Figure 6:**
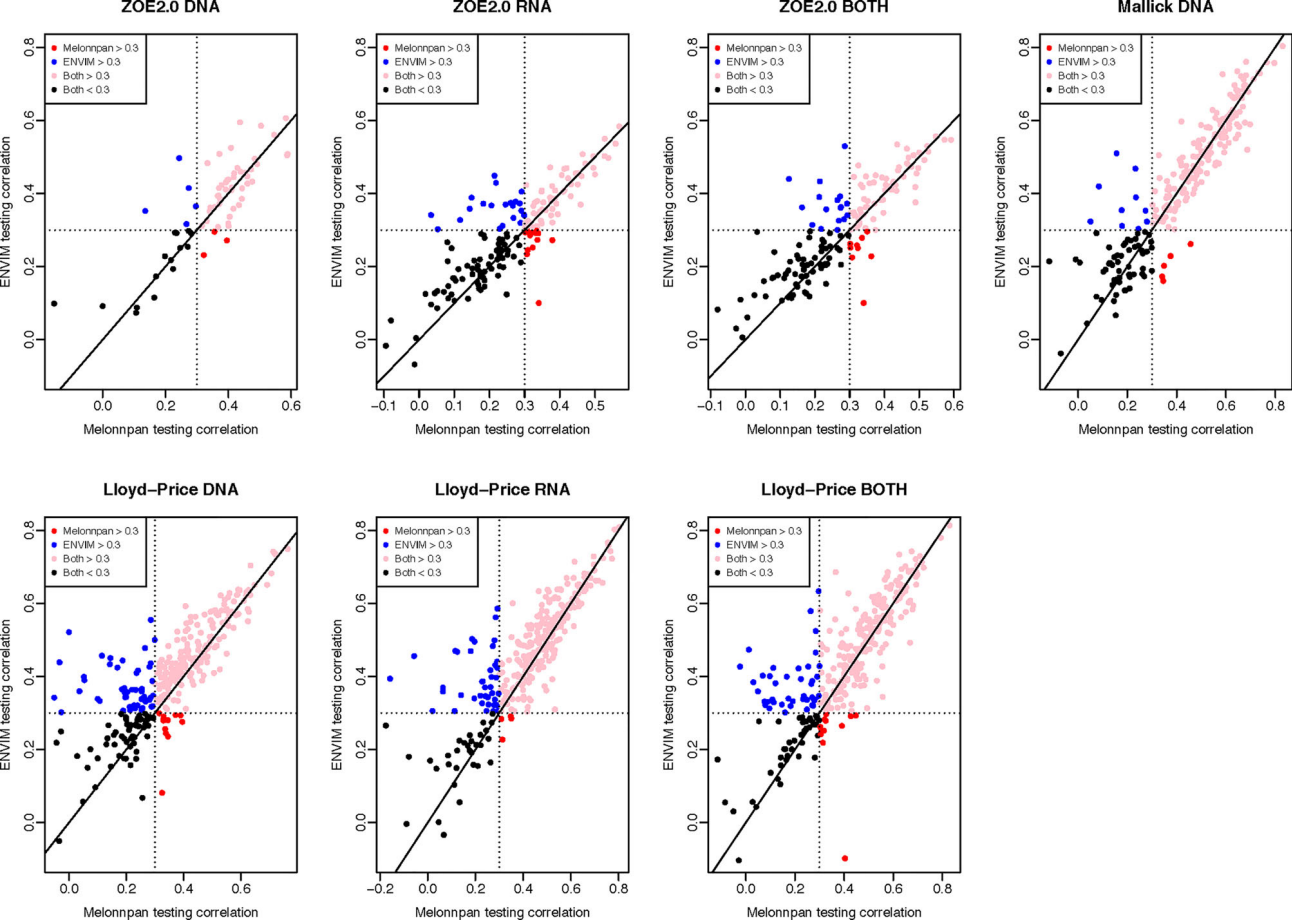
For DNA, RNA, and both in each study and the testing set, this shows the scatter plot of Spearman correlation in ENVIM (*y*-axis) and MelonnPan (*x*-axis). Spearman correlation is based on observed metabolite abundance and predicted values. Here, “Both ≥0.3” refers to the category of metabolites that have Spearman correlation ≥0.3 in both ENVIM- and MelonnPan-predicted results. “ENVIM ≥0.3” refers to the category of metabolites that have Spearman correlation ≥0.3 only between ENVIM-predicted and observed values.

Besides comparing MelonnPan and ENVIM in terms of percentages of well-predicted metabolites, one can directly compare the Spearman correlations of each predictable metabolite that is predicted by both methods (**Figures 5**, **6**). In the training set (**Figure 5**), for all three gene family data modalities and in all three datasets, we find that the majority of these metabolites have higher correlations in ENVIM compared with MelonnPan. The same holds in the testing set (**Figure 6**). We also observe that most points are along but slightly above the diagonal line in the testing sets (**Figure 6**). This suggests that the metabolites predicted by ENVIM have higher correlations with the observed ones compared with those predicted by MelonnPan. We also find that there are more metabolites in the “ENVIM ≥0.3” category (blue) than in the “MelonnPan ≥0.3” category (red). This is a reflection of more well-predicted metabolites found using ENVIM than using MelonnPan prediction.

To give a more realistic view of the improvement of the ENVIM over MelonnPan, as a tool to predict metabolites in practice, we use one of the two independent cross-sectional cohorts in Mallick data as training to predict the other. The PRISM cohort has 155 subjects and the NLIBD cohort has 65 subjects. For both ENVIM and MelonnPan, we use the microbiome and metabolome data in PRISM as the training set to predict the metabolites in NLIBD (**Table 3**). Among the 466 metabolites, ENVIM has 34% (160/466) in the testing set, while MelonnPan only has 26% (123/466) in the testing set. These percentages are very similar to 37% and 28%, respectively, in ENVIM and MelonnPan from random split of samples in the Mallick study, so that the same conclusion was drawn that better prediction power is in ENVIM than in MelonnPan.

**Table 3:** Prediction results in Mallick data, when using all samples in the PRISM study as the training set and the data in NLIBD study as the testing set.

Mallick cohort (NM = 466) PRISM (training, *n* = 155) NLIBD (testing, *n* = 65)	Training (ENVIM)	Training (MelonnPan)	Testing (ENVIM)	Testing (MelonnPan)
DNA only	387 (83%)	205 (44%)	160 (34%)	123 (26%)

To investigate the sample size effects, we further cut the sample size of the training set by half, or from 155 PRISM subjects to 77 or 78 subjects randomly for 10 times and find that with even half of the samples, ENVIM nearly maintains the well-predicted rates (**Table 4**). ENVIM is less sensitive to the decreased sample size than MelonnPan.

**Table 4:** Prediction results in Mallick data, when using half of the sample size in the PRISM study as the training set for 10 times and the data in NLIBD study as the testing set.

Mallick cohort (NM = 466) PRISM (training, *n* = 77 or 78) NLIBD (testing, *n* = 65)	Training (ENVIM)	Training (MelonnPan)	Testing (ENVIM)	Testing (MelonnPan)
Seed1	429 (92%)	161 (35%)	147 (32%)	96 (21%)
Seed2	402 (86%)	202 (43%)	162 (35%)	104 (22%)
Seed3	427 (92%)	164 (35%)	140 (30%)	92 (20%)
Seed4	428 (92%)	199 (43%)	148 (32%)	97 (21%)
Seed5	439 (94%)	211 (45%)	160 (34%)	111 (24%)
Seed6	427 (92%)	180 (39%)	157 (34%)	113 (24%)
Seed7	424 (91%)	178 (38%)	143 (31%)	98 (21%)
Seed8	424 (91%)	150 (32%)	142 (30%)	98 (21%)
Seed9	425 (91%)	159 (34%)	150 (32%)	101 (22%)
Seed10	419 (90%)	181 (39%)	152 (33%)	105 (23%)
Mean	424 (91%)	179 (38%)	150 (32%)	102 (22%)

#### Correlation-Based Method Comparison for Metabolites Within Metabolic Pathways

Metabolites may be associated with the microbiome in the context of metabolic pathways that involve interactions between the host, microbiome, and environment. We further investigate the predictive capability of the two methods for metabolites in MetaCyc metabolic pathways. HUMAnN 2.0 or 3.0 software provides information whether a MetaCyc metabolic pathway has been associated with microbiome data. In the MetaCyc database, we identify metabolites in each of these microbiome-associated pathways. All conclusions regarding method comparison, modality comparison, and body site comparison in the prediction of all metabolites still hold in the context of predicting metabolic-pathway-only metabolites. Additionally, when comparing the percentages of well-predicted metabolites among all metabolites (first four columns of **Table 2**) and those in the metabolic pathways (**Table 5**), we find higher predicted percentages for the latter.

**Table 5:** Prediction results *via* Spearman correlation for metabolites that are found in metabolic pathways.

	Training (ENVIM)	Training (MelonnPan)	Testing (ENVIM)	Testing (MelonnPan)
ZOE 2.0 (NM = 149)				
DNA only	129 (87%)	35 (23%)	57 (38%)	28 (19%)
RNA only	139 (93%)	73 (49%)	57 (38%)	40 (27%)
Both DNA and RNA	142 (95%)	73 (50%)	60 (40%)	46 (31%)
Mallick cohort (NM = 251)				
DNA only	231 (92%)	132 (53%)	94 (37%)	71 (28%)
Lloyd-Price cohort (NM = 125)				
DNA only	121 (97%)	71 (57%)	70 (56%)	40 (32%)
RNA only	125 (100%)	86 (69%)	103 (82%)	68 (54%)
Both DNA and RNA	124 (99%)	92 (74%)	105 (84%)	79 (63%)

Based on the criterion of Spearman correlation ≥0.3 between observed and predicted metabolites, we present the numbers of well-predicted metabolites with different prediction methods, datasets, and modality levels (DNA, RNA, and Both) and made a comparison between MelonnPan and ENVIM. NM is the number of metabolites to be predicted. Percentages in parentheses (%) represent the numbers of well-predicted metabolites divided by the total number of metabolites (NM) to be predicted in each study. The Mallick cohort has only metagenomics (DNA) data available and no pathway RNA data. The results from the Mallick cohort here are only based on filters (filtering out metabolites with mean relative abundance <10^−4^) and low prevalence (metabolites with >10% non-zero). In ZOE 2.0 and Lloyd-Price, metabolite data presented in this table have been selected according to membership in pathways and also satisfy the abovementioned filtering criteria.

#### MSE-Based Method Comparison

We use boxplots to compare MSE between measured and predicted metabolite abundances between ENVIM and MelonnPan both for training and testing models, with application to training and testing data for all three studies. We only compare well-predicted metabolites identified by MelonnPan in training, since MelonnPan only generates results for these metabolites. The boxplot demonstrates that the distribution of MSE in the MelonnPan model is approximately the same as the distribution of MSE in ENVIM (**Supplemental Figure 2**). We find no significant difference in MSE between ENVIM and MelonnPan, which suggests that both methods predict these metabolites well in terms of MSE. The advantage of ENVIM is that we can predict substantially more well-predicted metabolites than MelonnPan—a consequence of MelonnPan’s inability to build a well-performing model in the training step. When using PRISM as the training set and NLIBD as the testing set in the Mallick study, the above conclusion about MSE remains the same (**Supplemental Figure 3**).

### ENVIM Outputs Including Predicted Individual Metabolites and Contributing Gene Family Weights

#### Top Well-Predicted Metabolite Compounds From ENVIM

**Figure 7:**
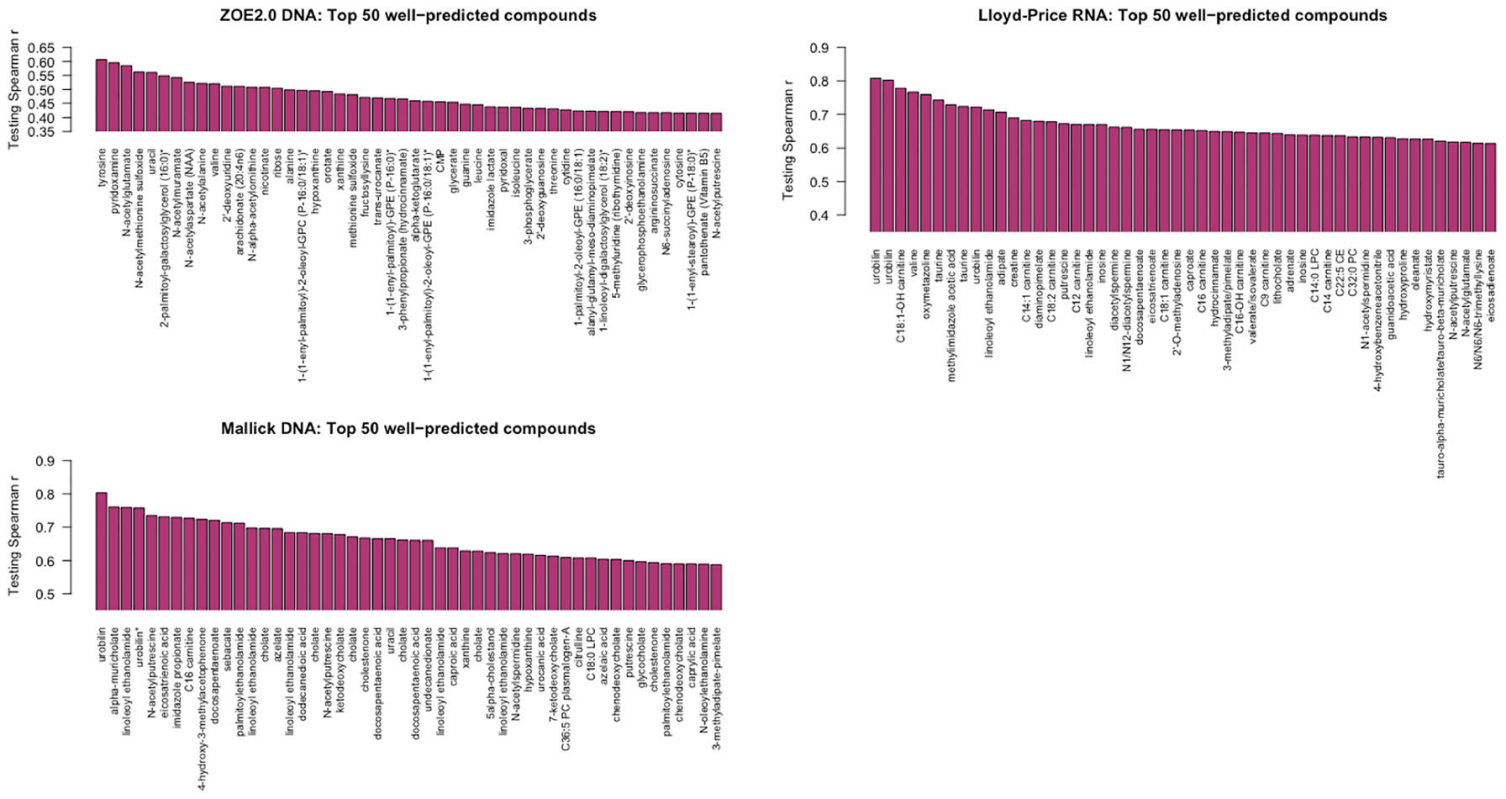
The best predicted 50 metabolite compounds (*x*-axis) in the three studies by ENVIM in the testing set. For Lloyd-Price and ZOE 2.0, we choose the gene family data types that have the best ENVIM prediction power to show their top predicted metabolites, based on **Table 2**.

For simplicity, we present one modality from each of the three studies. For Lloyd-Price and ZOE 2.0, we choose one of the gene family data modalities that has the best ENVIM prediction power to show their top predicted metabolites, that is, the DNA gene family data (124 metabolites as 25% among NM, **Table 2**) in ZOE 2.0 and the RNA gene family data (393 metabolites as 75% among NM, **Table 2**) in Lloyd-Price. Since the Mallick study only has DNA data available, the DNA gene family data are used. To note, both the Lloyd-Price study and the Mallick study have measured metabolites in four metabolome LC-MS platforms (see the *Cohorts and Data Description* section) so that one metabolite may appear multiple times in the top list (for example, urobilin). The top 50 best predicted metabolites for each study are presented in **Figure 7**.

**Figure 8:**
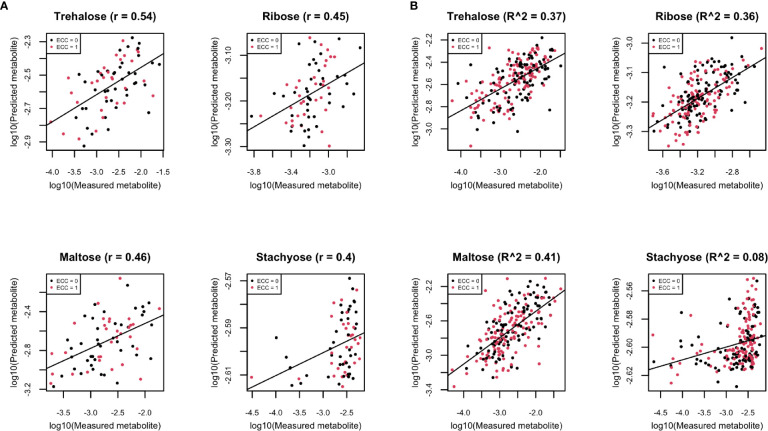
**(A)** Scatter plots of examples of four well-predicted metabolites in ZOE 2.0 by ENVIM, in the testing set. *r* is for Spearman correlation for method evaluation. **(B)** Scatter plots of the same four well-predicted metabolites in ZOE 2.0 by ENVIM, in the training set, where *R*-square (Pearson Correlation) was shown for the percentage of variance explained by prediction models to demonstrate that overfitting is not a big concern. The *x*-axis is the observed metabolites; the *y*-axis is the predicted metabolites. Both *x* and *y* are in log_10_ scale of the compositional data for normality. ECC stands for early childhood caries, ECC = 0 (about 50% of total samples in ZOE 2.0) is for the healthy group, and ECC = 1 (about 50% of total samples in ZOE 2.0) is for the ECC case group.

The summarized prediction results are presented in **Supplemental Table 1**. To interpret the results, we take the carbohydrate pathway as an example of a pathway that may provide bacteria with nutrition, which includes a few compounds that have been well-predicted by the RNA gene family data. We are aware that the prediction in this paper is not about longitudinal causal relation, but rather, for mathematical prediction. Here, we also show four examples (trehalose, maltose, ribose, and stachyose) that have high Spearman correlation on the log_10_ scale of the compositional data (**Figure 8A**).

#### Comparison of Gene Family Lists (With Weight Matrix) Across Three Datasets in ENVIM

We extract gene family names that have non-zero entries in the weight matrix for each metabolite, dataset, and gene family modality (**Supplemental Table 2**) in ZOE 2.0. We compare the contributing gene family names across the ZOE 2.0 and Lloyd-Price to find the number of common contributing genes the different body sites share for predicting metabolites. We find that there are not many overlapping genes (*n* < 10) between ZOE 2.0 data and Lloyd-Price data (data not shown).

#### Gene Set Enrichment Analysis of Contributing Genes Within Species in ZOE 2.0

We perform gene set enrichment analysis to find the over-represented species of the contributing gene families to predict metabolites in ZOE 2.0. To test that, we start with the weight matrix of gene families and metabolites in the testing set. We identify the contributing gene families that have non-zero values with any well-predicted metabolites.

**Figure 9:**
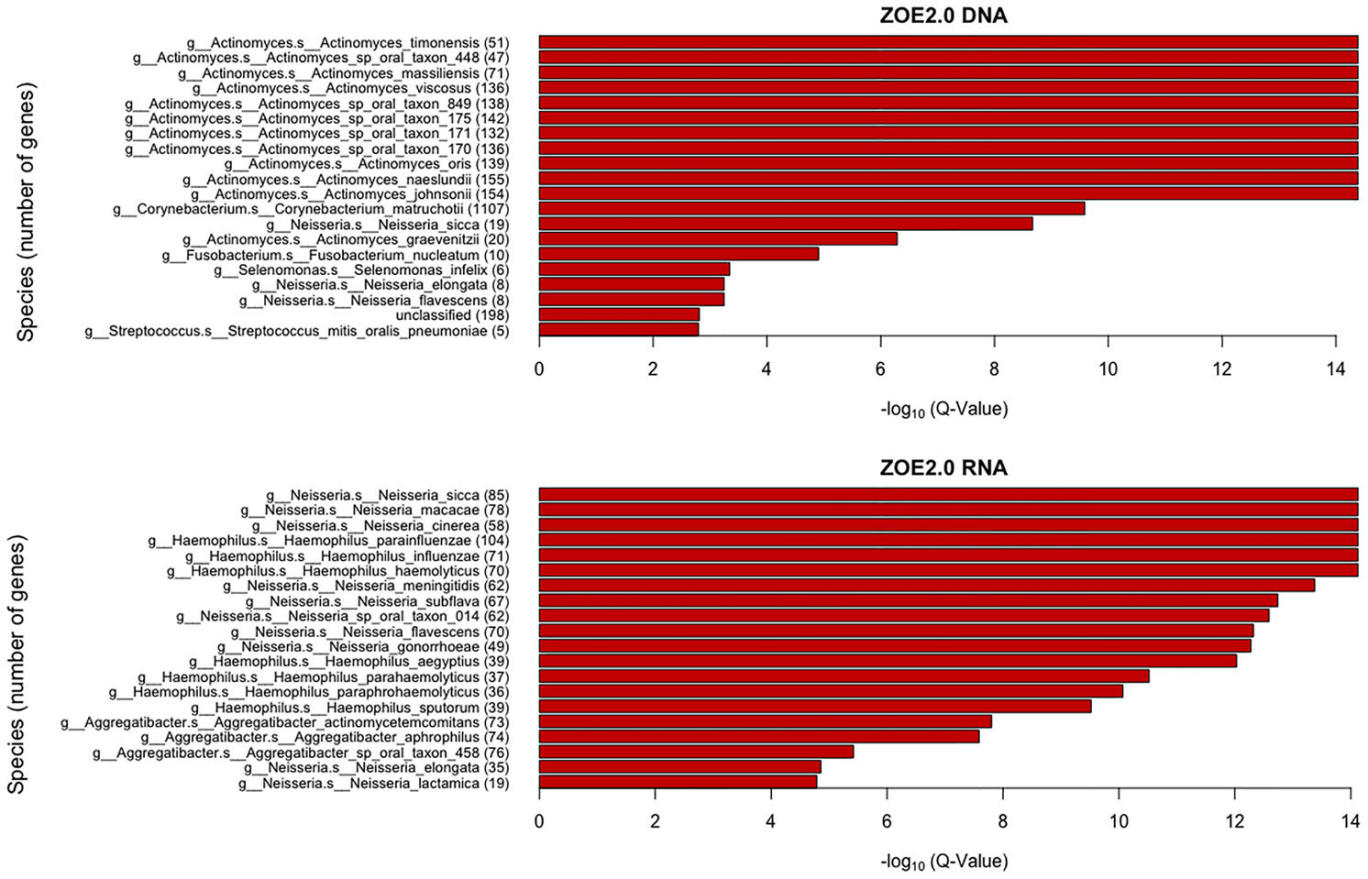
Taxonomic enrichment of metabolite predictive species for the most contributing species to metabolite prediction, based on ZOE 2.0 DNA or RNA by ENVIM. The top 20 significant over-represented bacteria with the smallest *Q*-values (*Q* < 0.05) for ZOE 2.0 data. The *Q*-value is based on the Kolmogorov–Smirnov (KS) test *p*-values after FDR correction. Upper, DNA data; Lower, RNA data.

We obtain the rank of each gene family in the weight matrix based on the absolute value of the regression coefficients (“weights”) for each gene family. We use the information of correspondence between gene families and the species level (generated in HUMAnN 2.0.) to identify the species corresponding to those contributing gene families. For each species, we compare the difference in the cumulative distributions of gene family rank scores between the species and the background species using the Kolmogorov–Smirnov (KS) test that was also used in the original gene set enrichment analysis (GSEA) paper (Subramanian et al., 2005). We use the Benjamini–Hochberg false discovery rate (FDR) approach to correct the KS *p*-values and get *q*-values. There are 36 species in ZOE 2.0 DNA data and 73 species in ZOE 2.0 RNA data found to be significantly (*q* < 0.05) over-represented in the gene set enrichment analysis (**Figure 9**).

Here, we used a different procedure for the gene set enrichment tests compared to what was used in the MelonnPan (Mallick et al., 2019) paper, in that the gene families in genera instead of species were summarized as a gene set, due to the small number of gene families in each species in their prediction procedure. In fact, ENVIM keeps many more genes than MelonnPan (because ENVIM allows larger range of *α*) so that ENVIM can address the ranks of all contributing gene families instead of the binary prediction power of genes (i.e., whether a gene is used for prediction or not) used in MelonnPan and, furthermore, can perform GSEA at the species level for higher resolution of contributing species. Our GSEA strategy also can help avoid the bias of selecting for species that have larger numbers of genes.

### Computational Speed

ENVIM was implemented in R statistical language. It can accurately predict metabolites using matched microbiome gene family data. The mean running time in ENVIM of each metabolite using DNA gene family data is 5.2 min for ZOE 2.0 data (6.1 min for Lloyd-Price data, 2 min for Mallick data). The mean running time in ENVIM using RNA gene family data is 4.2 min for ZOE 2.0 data (3.7 min for Lloyd-Price data); the mean running time in ENVIM for both DNA and RNA gene family data is 4.5 min for ZOE 2.0 data (3.6 min for Lloyd-Price data) with MacOS Big Sur Version 11.4 and the desktop iMac Pro 2020.

## Discussion

We propose a new computational method for metabolite prediction using microbiome data-based improved elastic net models. We chose different gene family sets based on random-forest-based variable importance scores and modified the existing ENM to accommodate the unique features of microbiome and metabolome data. The newly developed method ENVIM predicts metabolites using metagenomics, metatranscriptomics, or both data types. We apply the algorithm in three datasets, i.e., the ZOE 2.0, Mallick, and Lloyd-Price studies. These three studies have both microbiome and metabolome data in the same matched samples, with reasonably large sample sizes. We are the first to use microbiome data to predict metabolites in more than one study and different body sites. In addition, the ZOE 2.0 and Lloyd-Price studies have both metagenomics and metatranscriptomics, so that we can, for the first time, compare prediction performance using the different gene family modalities (or called data types).

We evaluated metagenomic and metatranscriptomic predictors and compared the prediction performance between the previously developed MelonnPan and ENVIM, among DNA, RNA, and both DNA and RNA gene family data using 1) the proportion of “well-predicted” metabolites defined as those with Spearman correlation between measured and predicted metabolite values ≥0.3, 2) distribution of Spearman correlation, and 3) MSE. The correlation suggests that Both (of DNA and RNA) provides robust prediction results that are never the worst among the three data types. Whether DNA or RNA has better prediction performance depends on the study. The percentage of well-predicted metabolites is higher for metabolites that are in a metabolic pathway observed in the microbiome data. Such enrichment of well-predicted metabolites in metabolic pathways supports the strong interaction between microbiome and metabolome. Across all datasets and data types, with or without the pathway filter, we find that ENVIM always outperforms MelonnPan. We also find that prediction performance is better in Lloyd-Price and Mallick than in ZOE 2.0, which suggests that the association between microbiome and metabolites is stronger in the gut than in the oral cavity, since oral metabolites may be more affected by environmental factors like food intake. More microbial omics studies are needed to compare the prediction power across different body sites and to understand how the microbiome interacts with the metabolome differently at different body sites. Acknowledging that the reported findings are not to infer causality but are demonstrative of mathematical prediction, we show four well-predicted metabolites in ZOE 2.0 (**Figure 8**), as examples of compounds that may play roles in bacterial metabolism.

As a result, the numbers of the measured metabolites and the numbers of the to-be predicted metabolites in each of the three studies are very different due to differences in technology platforms, data processing steps, and available data at different body sites. Besides body sites, the data collection and processing steps may have large effects on the prediction performance. The distributional assumption, normalization, transformation, outlier filtering, and missing data handling are important considerations before training the model. We have touched on that, but further exploration may be needed. According to what we observed, the ideal usage of these types of prediction methods is in studies that contain paired microbiome and metabolome data in one batch of samples but lack metabolome data in other batches of samples. In that case, all microbiome samples are sequenced, aligned, and processed comparably, and the uncollected metabolome samples are also assumed to be from the same technical metabolome platform and similar data processing steps (for example, as what we demonstrated in **Tables 2**, **5**). However, the usage of these methods is not limited only to this ideal case. The suitable usage scope has the assumptions of 1) the same population distribution of microbiome data in the training model and in the cohort to be predicted, 2) the same population distributions between the metabolome data in the training model and in the cohort to be predicted, and 3) similar connection between microbiome and metabolome due to, for example, similar ethnicities, clinical characteristics, age groups, and body sites (as shown in **Table 3**). Usage of the IBD Lloyd data to predict metabolites in the IBD Mallick study *via* ENVIM and MelonnPan has been considered. Although these two studies have been generated from the same body sites and similar LC-MS metabolome techniques, their microbiome data have been processed in different versions of HUMAnN software (3.0 vs. 2.0), and in different data scales (CPM vs. RPKM), their metabolome data have been processed using different algorithms in different software, and different filtering criteria have been used in the two studies. These differences suggest that the first and the second assumptions are not held well, and the prediction results are not encouraging (data not shown). Furthermore, the assumptions of similar population distributions depend on the measuring technical platforms, the data processing steps, and proper normalization methods. The questions of what the best normalization method is and which integration procedure best allows assumptions of similar population distributions to overcome the difference across cohorts/technique/data processing are out of the scope of this paper but are very important for further data harmonization of microbiome-related large datasets.

Because higher well-predicted metabolite rates were observe in the training compared to the testing datasets, overfitting of the machine learning model can be a concern; however, overfitting is not a great concern around ENVIM for the following reasons: 1) similar observed mean square error in the training set and the testing set (**Supplemental Figures 2**–**4**) and 2) small squared Spearman correlation (*R*-square) between fitted and observed metabolites in the training sets (**Figure 4B**). Four well-predicted metabolites in ZOE 2.0 have no large *R*-square in the training set, and similar patterns in the scatter plots between measured and predicted metabolites are observed in both the training set and testing set (**Figure 8B**), and 3) the penalty terms in ENM, cross-validation in tuning the penalty terms, and the use of bootstrapping in random forest relax the potential overfitting problem. Although the overfitting concern is reasonably mitigated, it should be acknowledged that it may not be perfectly avoided. With that in mind, the method performance in the testing set is the most important. We observe that ENVIM has a higher well-predicted metabolite percentage (**Tables 2**, **3**, **5**) and comparable MSE (**Supplemental Figures 2**, **3**) when compared with MelonnPan.

A limitation in the framework for ENVIM, as well as in the framework for MelonnPan, is that the experimental design in studies, including time course or disease statuses, has yet to be considered. However, since the purpose of ENVIM is prediction, the prediction does not need to be conditional on the experimental design. Instead, different disease statuses may have different microbiome profiles and, correspondingly, have different metabolome profiles. Therefore, the non-inclusion of a design matrix in ENVIM is a limitation but not a drawback of the prediction performance.

In summary, we illustrate that the newly developed ENVIM method for microbiome-based metabolite prediction provides good prediction performance and can be used to predict individual metabolites when only microbiome data are available if the same technical microbiome/metabolome platform, similar data processing steps, and the same body site and covariate values can be assumed, or when a proportion of samples in a study have no metabolome data.

## Data Availability Statement

The ZOE 2.0 microbiome data have been deposited in dbGaP under the umbrella Trans-Omics for Precision Dentistry and Early Childhood Caries or TOPDECC (accession: phs002232.v1.p1). Metabolomics raw spectral data and associated clinical traits have been deposited in the MetaboLights repository: https://www.ebi.ac.uk/metabolights/MTBLS2215. Descriptive and clinical data for the parent ZOE 2.0 study have been deposited in https://doi.org/10.17615/8yjy-w790.

## Ethics Statement

Ethics approval was received by the Institutional Review Board of UNC-Chapel Hill (#14-1992) for the ZOE 2.0 study; all participants’ guardians provided written informed consent for participation in the ZOE 2.0 study. For the Lloyd-Price and Mallick cohorts, the data we have used are publically available on line. Therefore, "ethical review and approval was not required for the study on human participants in accordance with the local legislation and institutional requirements. Written informed consent from the participants’ legal guardian/next of kin was not required to participate in this study in accordance with the national legislation and the institutional requirements."

## Author Contributions

Conceptualization by DW and JX. Supervision by DW. Investigation by JX and LH. Formal analysis by JX. Writing—original draft preparation by JX, HC, BL, MP, KD, and DW. Reviewed draft also by FZ. Discussion with DB and FZ. All authors contributed to the article and approved the submitted version.

## Funding

We acknowledge NIH/NIDCR R03-DE02898, NIH/NIDCR U01-DE025046, P30 CA016059 (Massey Cancer Center Support Grant) for funding support, and NLM T15 training grant #T15-LM012500 for funding support.

## Conflict of Interest

The authors declare that the research was conducted in the absence of any commercial or financial relationships that could be construed as a potential conflict of interest.

## Publisher’s Note

All claims expressed in this article are solely those of the authors and do not necessarily represent those of their affiliated organizations, or those of the publisher, the editors and the reviewers. Any product that may be evaluated in this article, or claim that may be made by its manufacturer, is not guaranteed or endorsed by the publisher.

## Supplementary Material

Supplementary Figure 1 Diagnosis for outlier samples in metabolome data. The x-axis is the cumulative proportion of samples, and the y-axis is number of non-missing values. The left lower tail dots that are far from the rest may be considered as sample outliers. For ZOE 2.0 data and Lloyd-Price data, we need to remove the 10 outliers subjects from ZOE 2.0 data and 15 outliers from Lloyd-Price data to ensure the distribution of non-missing values is continuous.

Supplementary Figure 2 Boxplot of -log_10_ of MSE for DNA, RNA, and BOTH in each of the three studies to compare ENVIM and MelonnPan.

Supplementary Figure 3 Boxplot of -log_10_ of MSE for DNA, RNA, and BOTH in Mallick study when PRISM data was used as training to predict metabolites in NLIBD data. This is to compare ENVIM and MelonnPan.

Supplementary Figure 4 Boxplot of -log_10_ of MSE for DNA, RNA, and BOTH in each of the three studies, for all metabolites predicted by ENVIM.

Supplementary Table 1 Overall prediction results, for all gene family data types, all three datasets, and both methods, in Spearman correlation and MSE.

Supplementary Table 2 The gene lists in DNA or RNA, based on the highest rank or the average rank among metabolites, that contribute to prediction of well-predicted metabolites in ZOE 2.0 by ENVIM. Rank is based on the weight matrix in ENVIM. A larger number of ranks suggests more important gene families.
