# Supplementary material for: Improved Metabolite Prediction Using Microbiome Data-Based Elastic Net Models
Source: Front Cell Infect Microbiol. 2021 Oct 25;11:734416. doi: 10.3389/fcimb.2021.734416 (PMC8573316; doi:10.3389/fcimb.2021.734416)
Supplement: Supplementary Figure 1 — Diagnosis for outlier samples in metabolome data. The x-axis is the cumulative proportion of samples, and the y-axis is number of non-missing values. The left lower tail dots that are far from the rest may be considered as sample outliers. For ZOE 2.0 data and Lloyd-Price data, we need to remove the 10 outliers subjects from ZOE 2.0 data and 15 outliers from Lloyd-Price data to ensure the distribution of non-missing values is continuous. [file Image_1.pdf]

Sup-Figure 1

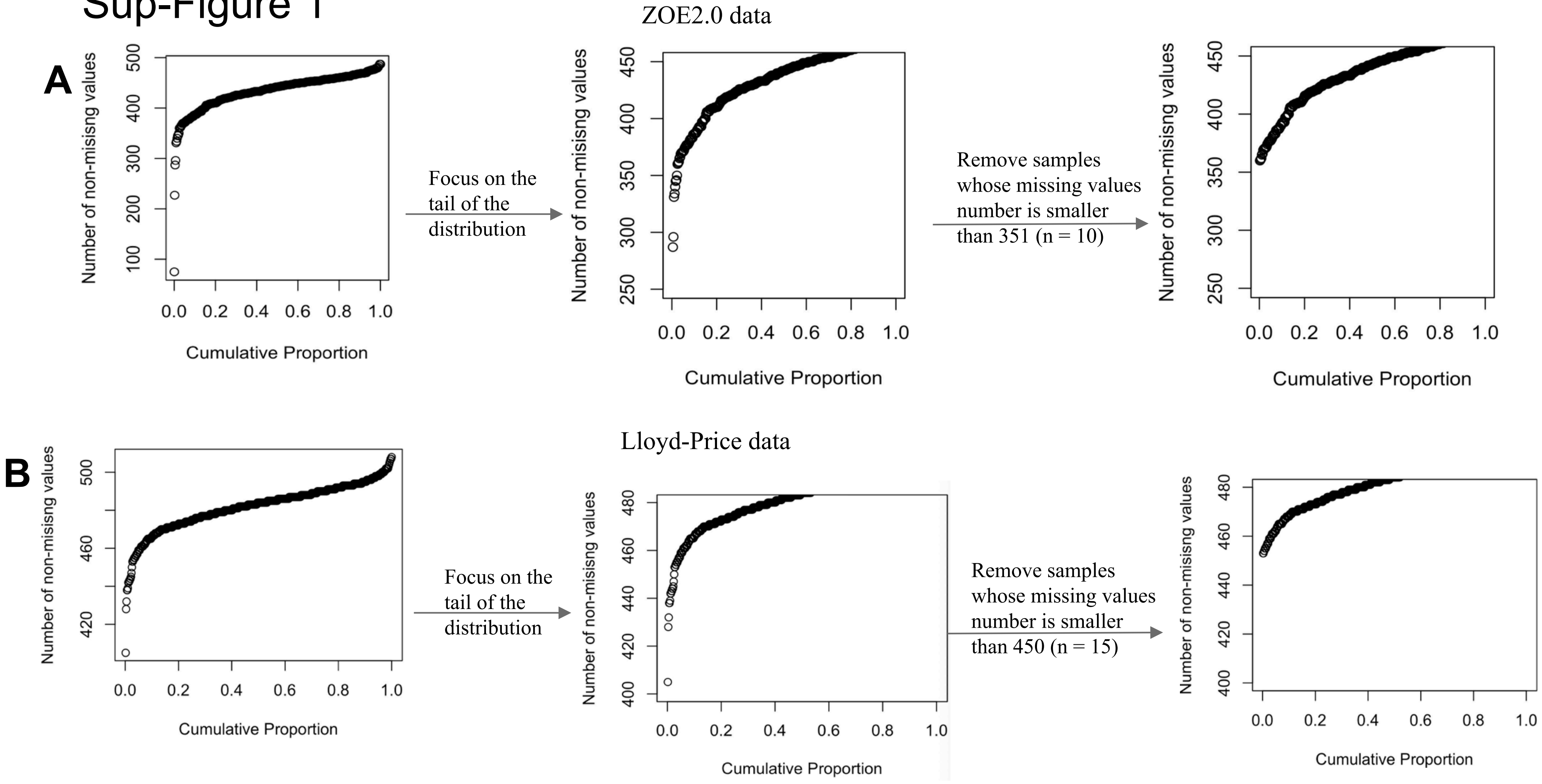
ZOE2.0 data
A
Number of non-misisng values
Cumulative Proportion
Focus on the tail of the distribution
Remove samples whose missing values number is smaller than 351 (n = 10)
Lloyd-Price data
B
Focus on the tail of the distribution
Remove samples whose missing values number is smaller than 450 (n = 15)
