# Supplementary figures and images for: Improved Metabolite Prediction Using Microbiome Data-Based Elastic Net Models
Source: Front Cell Infect Microbiol. 2021 Oct 25;11:734416. doi: 10.3389/fcimb.2021.734416 (PMC8573316; doi:10.3389/fcimb.2021.734416)

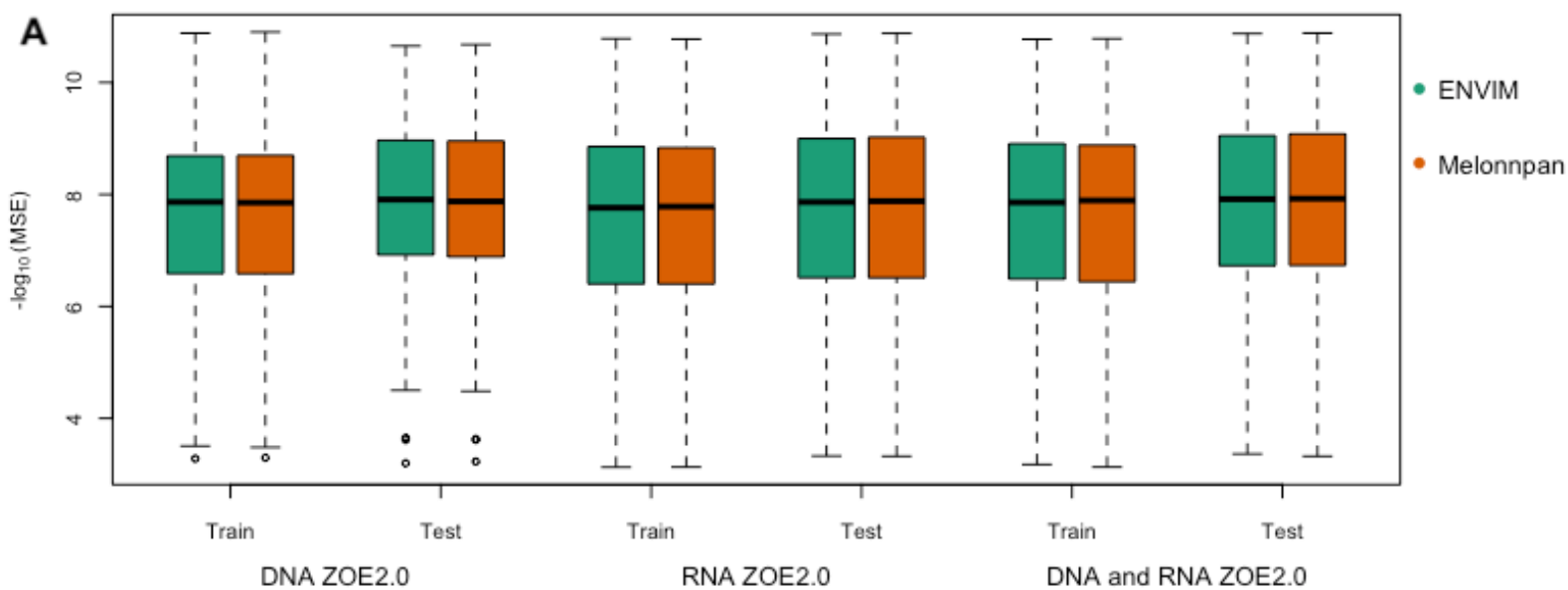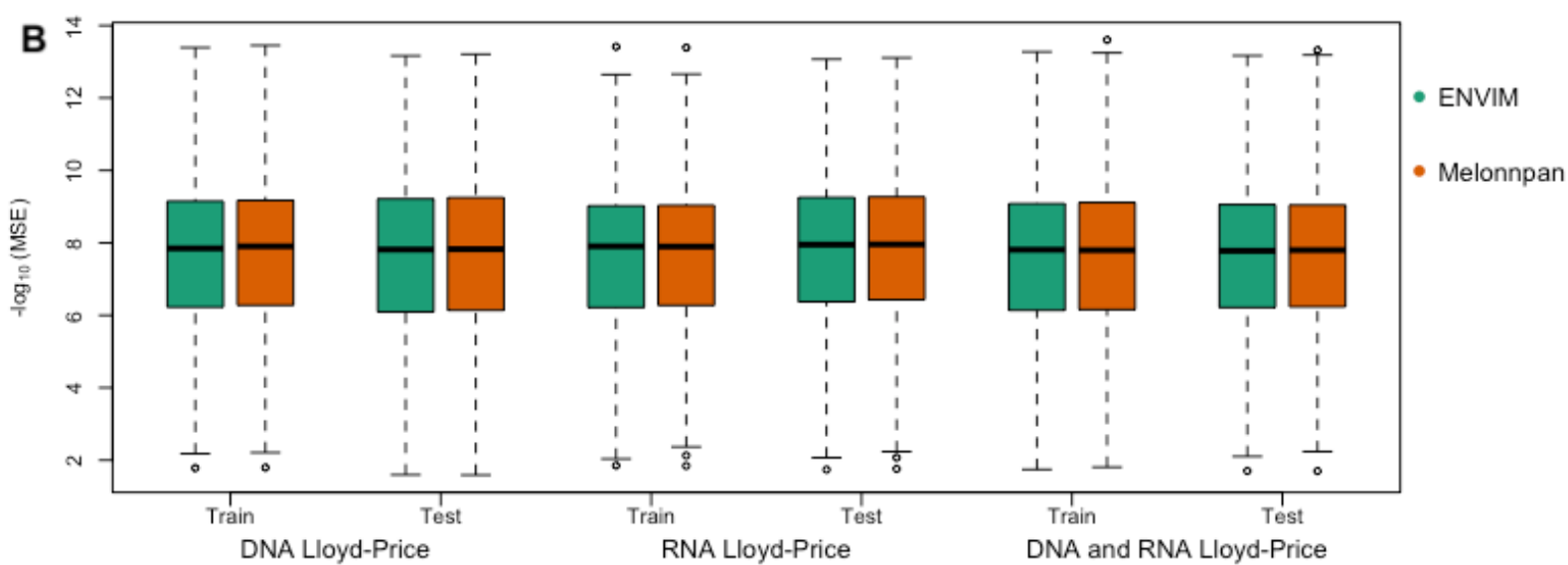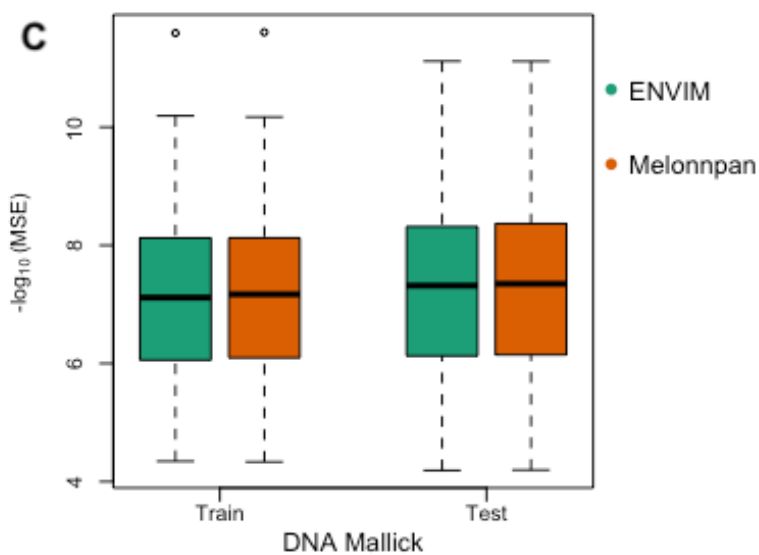

Supplement: Supplementary Figure 2 — Boxplot of -log10 of MSE for DNA, RNA, and BOTH in each of the three studies to compare ENVIM and MelonnPan. [file Image_2.pdf]

# Mean Square Error

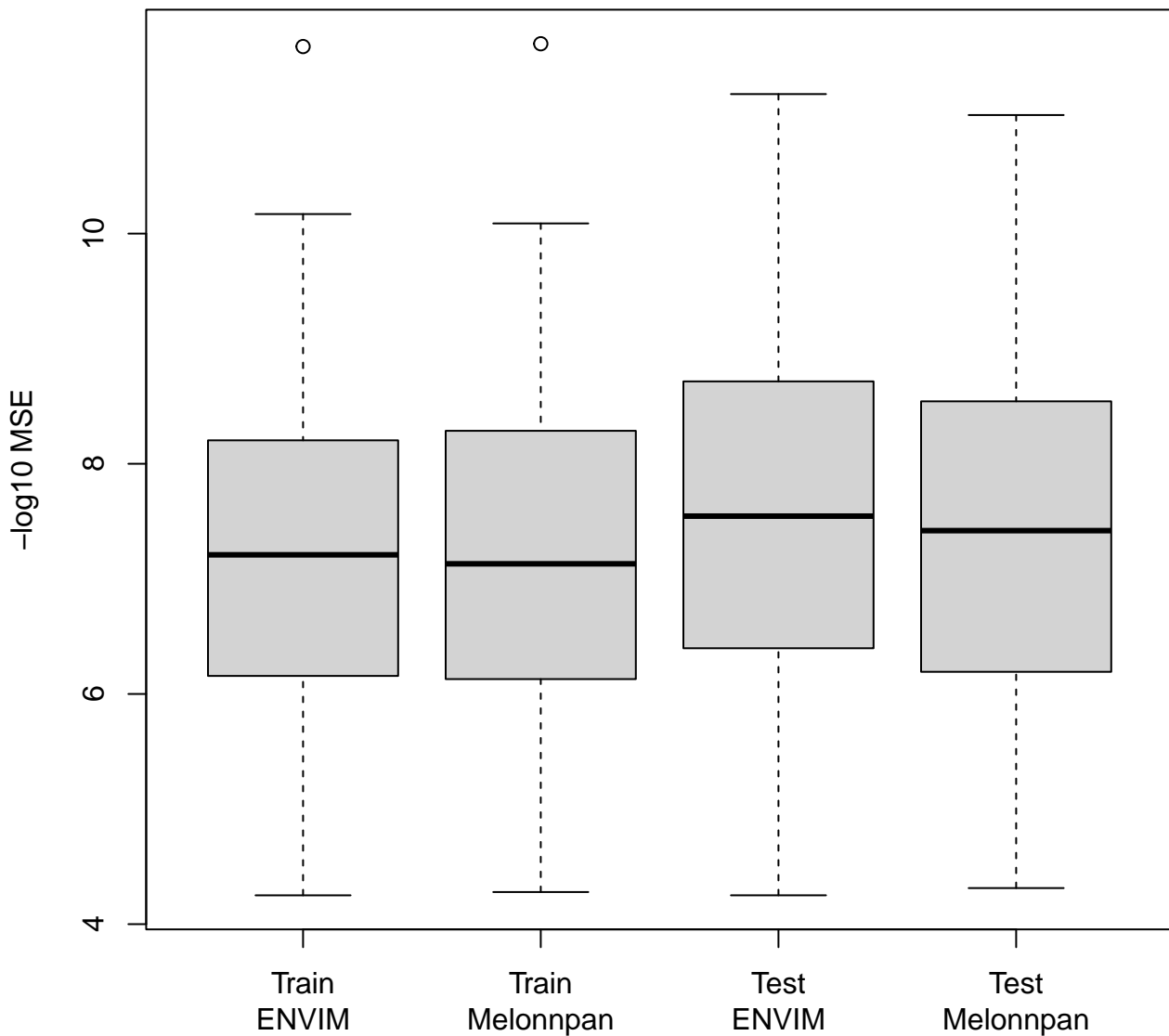

Supplement: Supplementary Figure 3 — Boxplot of -log10 of MSE for DNA, RNA, and BOTH in Mallick study when PRISM data was used as training to predict metabolites in NLIBD data. This is to compare ENVIM and MelonnPan. [file Image_3.pdf]

## ZOE2.0

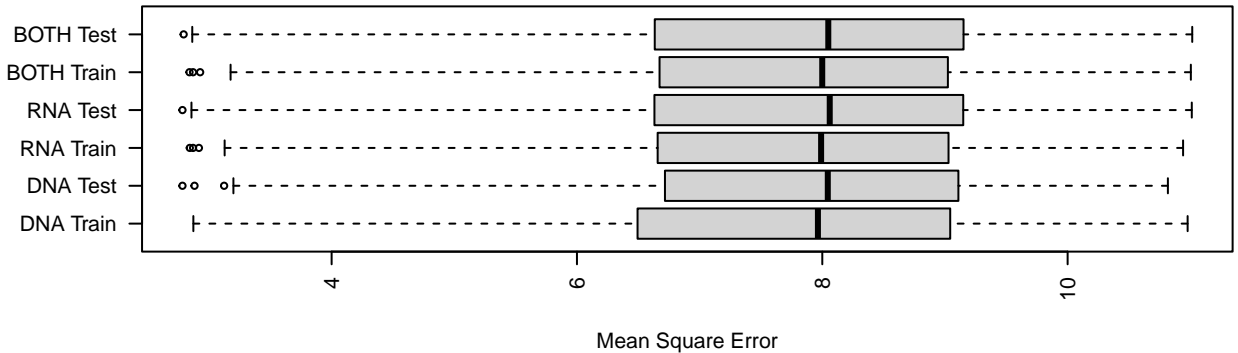

## Lloyd-Price

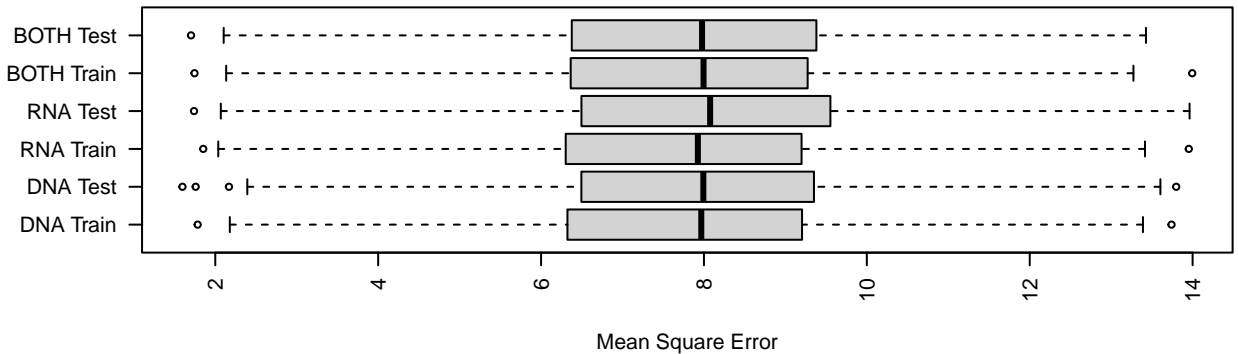

## Mallick

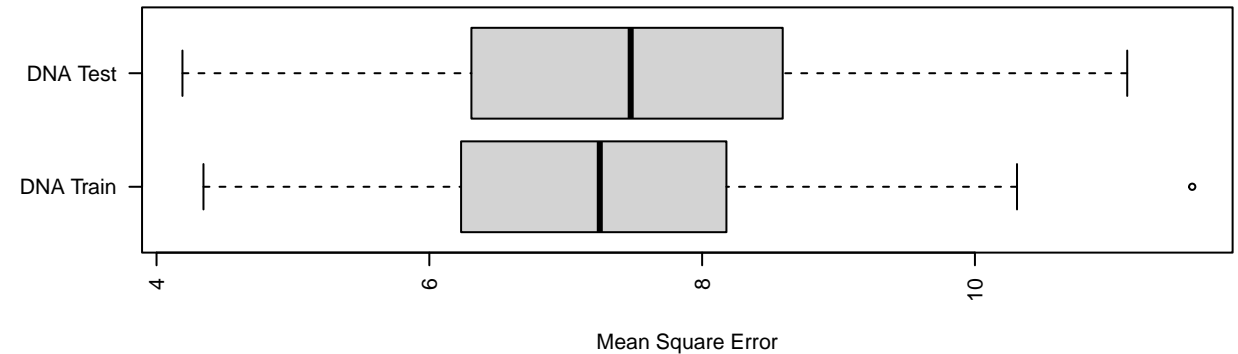

Supplement: Supplementary Figure 4 — Boxplot of -log10 of MSE for DNA, RNA, and BOTH in each of the three studies, for all metabolites predicted by ENVIM. [file Image_4.pdf]
